# Effects of meteorological factors on the incidence of *meningococcal* meningitis

**DOI:** 10.4314/ahs.v17i3.25

**Published:** 2017-09

**Authors:** Xue Bai, Bingxue Hu, Qi Yan, Ting Luo, Bo Qu, Nan Jiang, Jie Liu, Yaxin Zhu

**Affiliations:** School of Public Health, China Medical University, Shenyang, Liaoning Province, 110013, China

**Keywords:** *Meningococcal* meningitis, *Neisseria meningitidis*, epidemiology, humidity, temperature, sunshine, meteorological variables, structure equation model

## Abstract

**Background and Objectives:**

Substantial climate changes have led to the emergence and re-emergence of various infectious diseases worldwide, presenting an imperative need to explore the effects of meteorological factors on serious contagious disease incidences such as that of *meningococcal* meningitis (MCM).

**Methods:**

The incidences of MCM and meteorology data between 1981 and 2010 were obtained from Chaoyang city. Structure Equation Modeling was used to analyze the relationships between meteorological factors and the incidence of MCM, using the LISREL software.

**Results:**

The SEM results showed that Adjusted Goodness of Fit Index (AGFI) = 0.30, Goodness of Fit Index (GFI) = 0.63, and Root Mean Square Error of Approximation (RMSEA) = 0.31. Humidity and temperature both had negative correlations with MCM incidence, with factor loads of −0.32 and −0.43, while sunshine was positively correlated with a factor load of 0.42. For specific observable variables, average air pressure, average evaporation, average air temperature, and average ground temperature exerted stronger influence, with item loads between observable variables and MCM incidence being −0.42, 0.34, −0.32, and −0.32 respectively.

**Conclusion:**

Public health institutions should pay more attention to the meteorological variables of humidity, sunshine, and temperature in prospective MCM control and prevention.

## Introduction

*Meningococcal* meningitis (MCM) is a global respiratory infectious disease caused by *Neisseria meningitidis*, with the sudden onset of headache, fever, neck stiffness, vomiting and photophobia.^1^,^2^ The infection is spread from person to person via respiratory droplets.^2^ It is widely acknowledged that MCM remains a serious health issue globally, with an estimation of 1.2 million infections and 135,000 deaths per year.^3^ MCM incidences are high in developing countries, especially in the African meningitis belt, a region in sub-Saharan Africa, which extends from Senegal in the West to Ethiopia in the East.^4^ This region is characterized by the highest incidence of *meningococcal* disease in the world and experiences frequent epidemics causing major public health burden.^5^ Serious MCM there could affect up to 1% of the population during epidemics and have resulted in hundreds of thousands of cases and over 25,000 deaths between 1996 and 1997 alone.^5^ Major MCM epidemics have also occurred in large parts of Asia, such as in India in 2005–2006^6^ and Philippines in 2004–2005.^7^ Moreover, an annual disease rate of up to 500 cases per 100,000 people, resulted in several million cases of meningococcal disease in China between 1950 and 1980.^8^ Despite improvements in sanitation and health awareness, control measures have been met with limited success, and MCM remains a serious disease.^9^

MCM is a contagious disease which is not only influenced by microbial factors which affect the virulence of the microorganism but also by environmental factors.^3^ Currently, substantial weather changes are exerting a significant impact on disease transmission. In the year 2000, an estimated 150,000 deaths worldwide, caused by diseases such as increased diarrhea and respiratory diseases, have been related to climate change.^10^ There is an increasing need to assess the potential impact of climate change on MCM. Therefore, many studies have been conducted on the influences of different meteorological variables on the transmission of air-borne diseases like MCM.^11^–^13^ The seasonality of MCM outbreaks has already been found in Western Africa and China.^11^,^13^,^14^ In addition, various meteorological variables such as humidity, temperature, wind, and sunshine, were confirmed as important risk factors of MCM occurrence.^15^,^16^

Although the relationships between meteorological variables and MCM incidence have already been researched before,^17^–^19^ there still lacks studies on the comprehensive effects and quantification of the risk factors. The quantitative and comprehensive assessment of influencing factors on MCM plays a key role in the strategies, policies, and measures to control MCM, as recommended by the World Health Organization.^13^ It is highly important for us to perform comprehensive and quantitative analyses on the effects of meteorological variables, such as temperature, humidity, pressure, and sunshine, in order to understand the relationships between meteorological factors and the incidence of MCM. Therefore, the present study aimed to use SEM to explore the quantitative relationships between several meteorological variables and MCM incidence.

## Method

### Study area

Located in the Western part of Liaoning province, Chaoyang city lies at latitude 40′25″ - 42′22″ North and longitude 118′50″ - 121′17 East. It typically belongs to the Northern temperate continental monsoon climate, with four distinctive seasons. In summer it is warm and rainy, while in winter it is cold and dry. Its annual average temperature is 5.4 degrees Celcius. The annual average sunshine time is from 2850 to 2950 hours, and the annual average precipitation ranges between 450 and 580 millimeters.

### Data collection

The time frame of this study was set from 1981 to 2010, in total 360 months. Data of monthly incidence of meningitis were obtained from the Center for Disease Control and Prevention of Chaoyang city. All cases included in our study were confirmed by excluding the suspected MCM cases in advance.

The following monthly meteorological variables were collected from the Bureau of Meteorology of Chaoyang City. They mainly included monthly humidity indices: average evaporation, average air pressure, average monthly precipitation; monthly average temperature indices: maximum air temperature, average air temperature, minimum air temperature, maximum ground temperature, average ground temperature, minimum ground temperature; and monthly sunshine indices: average intensity of sunshine and average duration of sunshine.

### Data analysis

Due to the Northern temperate continental monsoon climate of Chaoyang city, some meteorological variables such as air temperature and air pressure have obvious annual cyclical change and show an inverted U-shaped trajectory. Logical errors were checked and corrected before the database was built. Pearson's correlation, using the Statistical Product and Service Solutions (SPSS® version 17.0, SPSS Inc., Chicago, IL, USA), was performed to find the correlation between monthly observed meteorological variables and monthly MCM incidence, with a lag of three months. SEM was applied to quantify the relationships between the incidence of MCM and latent meteorological variables. The linear structural relationships (LISREL) software (Scientific Software International, Lincolnwood, IL) was used to fit SEM.

## Results

### MCM incidence

**Figure 1:**
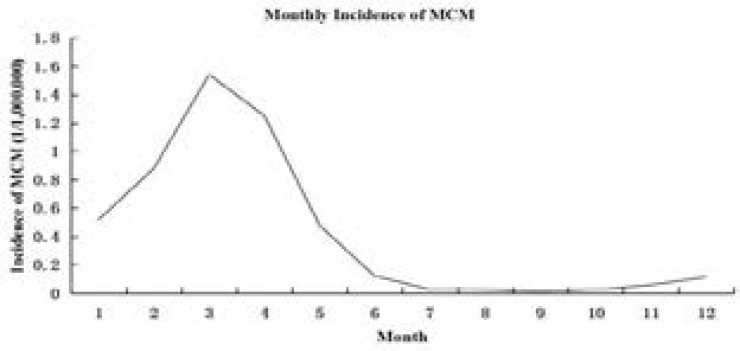
Monthly incidence of meningococcal meningitis (MCM) (Chaoyang City, China, 1981–2010)

Figure 1 shows that there was a clear seasonal variation in the incidence of MCM in Chaoyang city from 1981 to 2010, with most cases having been acquired in the late winter to early spring, starting generally from December, reaching a peak in March, and ending in July.

### Pearson's correlation analysis

Table 1 shows the correlation coefficients of specific observed variables to the monthly incidence of MCM. These statistically significant correlation coefficients ranged from −0.269 to 0.385 (P < 0.05). Prominently, air pressure, minimum air temperature, and maximum ground temperature were negatively related to the monthly MCM incidence, with correlation coefficients being −0.294, −0.269, and −0.263 respectively, while evaporation had a positive correlation with the incidence of MCM (a correlation coefficient of 0.385).

**Table 1:** Relationship between Meteorological Variables and MCM incidence (Chaoyang City, China, 1981–2010)

Meteorological Variables	Pearson correlation coefficients	Lag value (months)
Average air temperature	−0.239(**)	3
Maximumair temperature	−0.168(**)	3
Minimum air temperature	−0.269(**)	3
Ground temperature	−0.248(**)	3
Maximum ground temperature	−0.263(**)	3
Minimum ground temperature	−0.221(**)	3
Duration of sunshine	0.207(**)	3
Intensity of sunshine	−0.101	3
precipitation	0.076	3
Air pressure	−0.294(**)	3
Evaporation	0.385(**)	3

Note:

** Correlation is significant at the 0.05 level (2-tailed).

Lag 3: three months prior

Minimum air temperature, maximum ground temperature, and air pressure were negatively related to the monthly MCM incidence, with a lag of 3 months and relative high coefficients, while evaporation had a positive correlation with MCM incidence, with a lag of 3 months.

### Structure equation model

Structure equation model fitting results showed that the model was acceptable. Root Mean Square Error of Approximation (RMSEA) = 0.31, Goodness of Fit Index (GFI) = 0.63, and Adjusted Goodness of Fit Index (AGFI) = 0.30. The chi square value was 1150.68 (P < 0.05).

**Figure 2:**
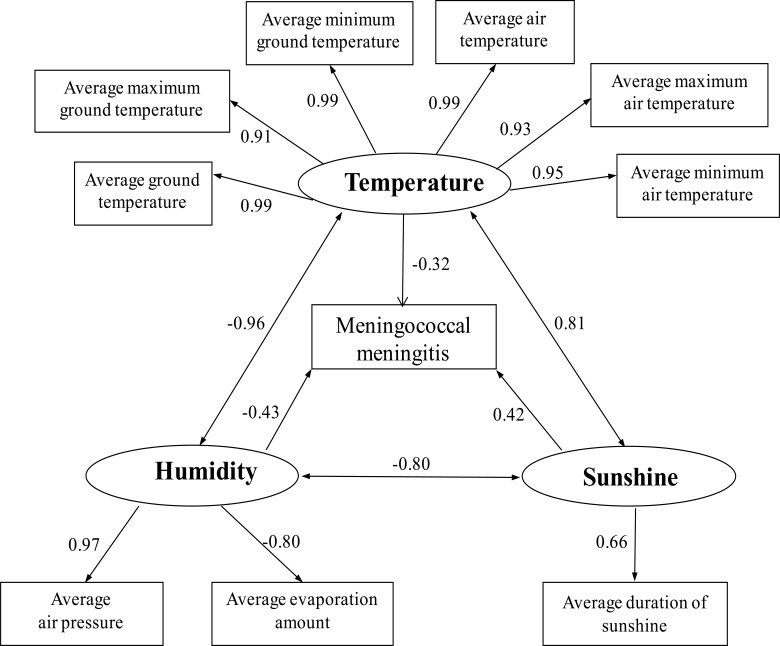
Significant pathways of the SEM and factors loads (ChaoyangCity, China, 1981–2010).

Figure 2 showed the association between the incidence of MCM which has been processed with a three-months lag, and various meteorological variables of humidity, temperature, and sunshine. The factor loads, −0.43, −0.32, and 0.42 in Figure 2 represented the association between the incidence of MCM and the three latent factors of humidity, temperature, and sunshine. This result suggested that humidity had the highest impact on meningitis occurrence. For a specific observable variable, the quantitative effect on the incidence of MCM was calculated by multiplying its item load by the factor load of the corresponding latent variable. In that way, average air pressure, average evaporation, average air temperature, and average ground temperature exerted stronger influence, with item loads between observable variables and MCM incidence being −0.42, 0.34, −0.32, and −0.32 respectively.

## Discussion

We found that MCM is a climate-sensitive infectious disease with a clear demonstration that the majority of disease occurrences concentrate in December to July of the next year, peaking in March. The indication of seasonality was generally consistent with other findings from different regions of the world.^13^,^15^ For meteorological variables, it was possible that ambient humidity, temperature, and sunshine, which had factor loads of −0.43, −0.32, and 0.42 respectively, not only affected the survival and transmission of *Neisseria meningitidis*, but also changed the activities and behaviors of the population, thus indirectly resulting in dynamics of the infection transmission.^20^,^21^

Compatible with previous studies in Italy^22^ and the African “meningitis belt”,^11^,^12^ the current study found a negative effect of humidity correlated with MCM incidence, with a factor load of −0.43. Additionally, average air pressure and average evaporation were highlighted with prominent effects, where each item had a load of 0.97 and −0.80 respectively. Consistent with local weather conditions, higher MCM incidence in Chaoyang city in winter is due to its typical Northern temperate continental monsoon climate with rare evaporation and high air pressure, which led to low humidity and facilitated the spread of the disease. Dry climates were reported to hold higher bacterial load in the air, resulting in more frequent *Neisseria meningitidis* transmission and facilitating the spread of the disease.^20^ Furthermore, dry air from low humidity could irritate the nasal mucosal barrier directly or inhibit mucosal immune defenses, enhancing *meningococcal* invasion.^23^ Mueller et al also found that higher humidity could favor the carriage of non-virulent *meningococci* and the reduction of MCM incidences.^24^

For temperature, we found that it was inversely related with MCM incidence, with a factor load of −0.32. In total, six observable factors were included in this latent variable. Among the six factors, average air temperature and average ground temperature exerted prominent influences on MCM transmission, both with item loads of 0.99. It was reported that *Neisseria meningitidis* replicates best at temperatures of 35°C–37°C,^3^ which is approximately the temperature of the human airways in a cold environment. In addition, breathing cold air causes cooling of the upper respiratory tract,^25^ and a recent study showed that cooling enhances the norepinephrine response of the nasal mucosa, which could indicate increased vasoconstriction in the upper airways, and the eventual depression of ciliary movements in the respiratory tract could increase susceptibility to infections.^26^,^27^ Moreover, it is also possible that people are at increased risk in winter due to the cold weather and dark evenings, making people tend to spend more time indoors and in closer proximity to others, which means transmission opportunity is increased with the easier spreading of pathogens.^21^

In comparison, a positive association between sunshine and MCM was displayed in our study, with a factor load of 0.42. Average duration of sunshine had a key impact on MCM incidence, with an item load of 0.66. The underlying reason might be that receiving sunlight during normal daily activities could suppress immunity in humans, 28 so infections may have spread to some extent due to low immunity. However, Kinlin et al found that exposure to ultraviolet B (UVB) radiation, which represents 5% of the total UV radiation present in sunlight,^29^ had a negative effect on *meningococcal* disease, with a dose-response relationship.^30^ So, the effect of sunshine on MCM incidence remains a question to be further explored in future studies.

In this study, all the correlated meteorological variables had a lag effect of 3 months on MCM. Time lag has been mentioned in many studies focused on relationships between meteorological factors and infectious disease.^31^–^33^ Peng Guan found that air pressure, temperature, and some other meteorological variables had correlations with incidence of hemorrhagic fever with a lag of 3 months, because virus incubation within the human body requires a certain period of time.^32^ The time lag would capture the period of virus incubation period within the human body and some other factors.^34^ In this study, Pearson's correlation was performed to quantify the relationships between meteorological variables and the incidence of MCM with the MCM incidence lagging from zero to three months, and the results of three months lag proved to be significant.

However, limitations to our study concerning representativeness should be acknowledged. Our data only came from a single city in China. In addition, we only focused on meteorological factors and did not take the *meningococcal* density and *meningococcal* antibody level in the population into consideration, which also exert important influences on MCM incidence. Therefore, further studies incorporating these factors should be conducted in order to provide a comprehensive guidance of controlling and preventing MCM epidemics.

## Conclusion

In summary, quantitative relationships exist between meteorological variables and MCM epidemics, where humidity and temperature both had negative correlations while sunshine had a positive correlation with MCM incidence. It is suggested that MCM prevention and control should give more consideration to meteorological variations for better understanding of MCM epidemics in the future.
